# Rapid eye movement sleep patterns of brain activation and deactivation occur within unique functional networks

**DOI:** 10.1002/hbm.25102

**Published:** 2020-06-23

**Authors:** Brandt D. Uitermarkt, Joel Bruss, Kai Hwang, Aaron D. Boes

**Affiliations:** ^1^ Neuroimaging and Noninvasive Brain Stimulation Laboratory, Departments of Pediatrics, Neurology & Psychiatry University of Iowa Hospitals and Clinics Iowa City Iowa USA; ^2^ Hwang Laboratory for Neurocognitive Dynamics, Department of Psychological & Brain Sciences University of Iowa Hospitals and Clinics Iowa City Iowa USA

**Keywords:** brain networks, fMRI, functional connectivity, magnetic resonance imaging, paradoxical sleep, REM sleep

## Abstract

Rapid eye movement (REM) sleep is a paradoxical state where the individual appears asleep while the electroencephalogram pattern resembles that of wakefulness. Regional differences in brain metabolism have been observed during REM sleep compared to wakefulness, but it is not known whether the spatial distribution of metabolic differences corresponds to known functional networks in the brain. Here, we use a combination of techniques to evaluate the networks associated with sites of REM sleep activation and deactivation from previously published positron emission tomography studies. We use seed‐based functional connectivity from healthy adults acquired during quiet rest to show that REM‐activation regions are functionally connected in a network that includes retrosplenial cingulate cortex, parahippocampal gyrus, and extrastriate visual cortices, corresponding to components of the default mode network and visual networks. Regions deactivated during REM sleep localize to right‐lateralized fronto‐parietal and salience networks. A negatively correlated relationship was observed between REM‐activation and deactivation networks. Together, these findings show that regional activation and deactivation patterns of REM sleep tend to occur in distinct functional connectivity networks that are present during wakefulness, providing insights regarding the differential contributions of brain regions to the distinct subjective experiences that occur during REM sleep (dreaming) relative to wakefulness.

## Graphical abstract

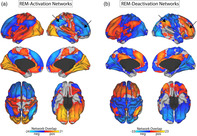

## 1 INTRODUCTION

The human brain entrains to a circadian rhythm that cycles through global states, each with distinct behaviors and subjective experiences. Most broadly this includes wakefulness, slow wave sleep, and rapid eye movement (REM) sleep. Among these states REM sleep tends to be the least well understood. REM sleep was initially noted due to its characteristic high frequency, low amplitude oscillations detectable on electroencephalogram (EEG) that resemble patterns seen in wakefulness (Aserinsky & Kleitman, 1953). REM sleep is conducive to dreaming, a state in which subjective experience is intrinsically generated during sleep. Dreaming can occur during any stage of sleep, but the highest likelihood of dreaming is during REM sleep, with vivid visual imagery and plots that include more complex content (Siclari et al., 2017). This differs from dream‐like mentation that can occur during wakefulness and from the thought‐like content that is more common with slow wave sleep (Cicogna, Natale, Occhionero, & Bosinelli, 2000; Fox, Nijeboer, Solomonova, Domhoff, & Christoff, 2013).

Most aspects of REM sleep are understood from animal models. There is evidence that acetylcholine is projected diffusely in the forebrain during REM sleep while monoamine systems are quiescent relative to wakefulness (Saper & Fuller, 2017). Much of the brainstem and hypothalamus circuitry that promotes REM sleep is understood in experimental animal models (Saper & Fuller, 2017; Schwartz & Roth, 2008). In humans the subcortical circuitry and large‐scale forebrain regions that may support REM sleep are less well‐understood. Multiple studies have shown regional activations and deactivations in REM sleep relative to wakefulness that span multiple brain regions (Braun et al., 1997; Fox et al., 2013). Electroencephalography has highlighted posterior brain areas that may have a preferential role in supporting dreaming during REM sleep (Siclari et al., 2017), though specific anatomical structures could not be resolved.

Despite progress in understanding the regional activation and deactivation patterns that occur during REM sleep, many questions remain. For example, do regional activation patterns tend to occur within a specific functional brain network associated with REM sleep (Domhoff & Fox, 2015)? Fox et al. (2013) observed that regional metabolic activations during REM sleep tend to occur within regions of the default mode network (DMN), but this has not been systematically investigated (Fox et al., 2013). Functional imaging studies have shown that the dynamic interactions between brain regions differ during REM sleep, so it is unclear whether patterns of altered metabolism during REM sleep would correspond to functional networks that can be observed during wakefulness (Chow et al., 2013). Similarly, do areas that are preferentially deactivated during REM sleep occur in random locations or are they part of a separate functional network? Decreased prefrontal cortex activity during REM sleep has been reported with some consistency (Maquet et al., 2005; Muzur, Pace‐Schott, & Hobson, 2002; Nofzinger, 1997). It has been hypothesized that decreased prefrontal activity during REM sleep may contribute to the lack of vigilance toward the environment or lack of reality monitoring that occurs with dreaming, but it is not clear whether REM deactivation occurs within functional networks implicated in these processes during wakefulness. Finally, it is not known whether a network relationship exists between sites of REM activation and deactivation. Taken together, we set out to provide a more comprehensive and systematic investigation of the network distribution of peak regional metabolic changes during REM sleep, including the connectivity pattern among sites of activation, deactivation, and between sites of activation and deactivation.

Here, we investigate whether regional activation and deactivation patterns observed during REM sleep occur within distinct, functionally connected networks, assessed from intrinsic patterns of blood oxygen level dependent (BOLD) activity. First, we systematically reviewed the literature to identify positron emission tomography (PET) studies reporting regions preferentially activated or deactivated during REM sleep compared to pre‐sleep wakefulness. We generated spherical regions of interest (ROIs) for each coordinate and used them to perform seed‐based resting state functional connectivity MRI (rs‐fcMRI). Importantly, the rs‐fcMRI data are from healthy adults resting quietly while awake, as opposed to studies that have evaluated rs‐fcMRI in individuals during REM sleep (Chow et al., 2013; Hong et al., 2009; Lövblad et al., 1999; Miyauchi, Misaki, Kan, Fukunaga, & Koike, 2009; Wehrle et al., 2005; Wu et al., 2012). We evaluate two main hypotheses: that regions of (a) REM‐activation and (b) REM‐deactivation each associate with distinct positively correlated functional networks. Thirdly, we investigate whether any pattern of functional connectivity exists between REM‐activation and REM‐deactivation regions.

## 2 METHODS

### 2.1 Identification of REM sleep activation and deactivation coordinates

We first searched the literature for PET studies that have reported regions associated with REM sleep compared to wakefulness. We searched PubMed using search terms (PET + REM + Dreaming + Rapid Eye Movement Sleep) in various combinations and reviewed the reference list for any studies that included imaging results for REM sleep versus wakefulness. Inclusion criteria included any primary or meta‐analytic PET studies that were sufficiently powered (*N* > 20) and compared REM sleep versus pre‐sleep wakefulness. We excluded any primary studies that included pre‐sleep tasks that attempted to modify REM activity patterns or studies that restricted the reported results to pre‐defined ROIs (Maquet et al., 2000). This search produced two primary studies (Braun et al., 1997; Eichenlaub et al., 2014) and two meta‐analyses (Fox et al., 2013; Jakobson, Laird, Maller, Conduit, & Fitzgerald, 2012). Braun et al. (1997) had 37 subjects and reported 10 activation ROIs and 7 deactivation ROIs; Eichenlaub et al. (2014) had 41 subjects and reported 18 activation ROIs and 11 deactivation ROIs; Fox et al. (2013) performed a meta‐analysis of 6 PET studies, 81 subjects, and reported 10 activation ROIs and 7 deactivation ROIs; Jakobson et al. (2012) performed a meta‐analysis of 10 PET studies, 78 subjects, and reported 5 activation ROIs and 5 deactivation ROIs. Because the Braun results were included in the Fox and Jakobson meta‐analyses we do not treat these as four independent cohorts, though Eichenlaub was not included in either meta‐analysis so those results are independent of the others.

Using these studies, we identified each peak coordinate reported for areas of activation or deactivation in association with REM sleep compared to pre‐sleep wakefulness. Coordinates from Braun, Fox, and Jakobson were reported in Talairach space so a nonlinear registration was computed between the Washington University 711‐2B Talairach atlas (Caret) and the MNI152 template using ANTs software (Avants et al., 2011; Van Essen, 2002). The resultant warp instructions were applied to Talairach coordinates to convert them into MNI152 atlas space. Eichenlaub's coordinates were reported in MNI152 space (http://nist.mni.mcgill.ca/?p=858, Grabner et al.,^2006^). A spherical ROI with a 6 mm diameter was created for each coordinate and used as a seed region in the generation of functional connectivity networks, as described below. Two activation peaks from the Fox meta‐analysis were excluded because 1) the reported Talairach coordinate was located within lateral ventricle before and after transformation to MNI152 space (Talairach: −6, 16, 10; MNI: −7, 12, 14) and 2) the reported coordinate was a duplicate to one of the reported coordinates from the Braun study (Braun et al., 1997; Fox et al., 2013). Coordinates for each ROI across all four studies can be found in Table S1 and their relative spatial distribution is depicted in Figure S1. We also classified all activation and deactivation coordinates in terms of their overlap with functional networks from a previously published atlas (Yeo et al., 2011). These data can be found in Table S2 and Figure S2.

### 2.2 Resting state functional connectivity MRI

The primary rs‐fcMRI dataset included 98 healthy right‐handed subjects (48 male subjects, age 22 ± 3.2 years), that were resting quietly at the time of data collection, and not in REM sleep. These data are part of a larger, publicly available data set used previously (Boes et al., 2015, 2018; Holmes et al., 2015). Rs‐fcMRI data were processed in accordance with previously described methods (Fox et al., 2005; Fox, Buckner, White, Greicius, & Pascual‐Leone, 2012; Fox, Halko, Eldaief, & Pascual‐Leone, 2012; Van Dijk et al., 2010). Participants completed two 6.2 min rs‐fcMRI scans during which they were asked to rest in the scanner (3T, Siemens) with their eyes open (TR = 3,000 ms, TE = 30 ms, FA = 85°, 3 mm voxel size [27 mm^3^], FOV = 216, 47 axial slices with interleaved acquisition and no gap). Functional data were acquired at 3 mm voxel size (27 mm^3^) and spatially smoothed using a Gaussian kernel of 4 mm full‐width at half‐maximum. The data were temporally filtered (.009 Hz < *f* < .08 Hz) and several nuisance variables were removed by regression, including the following: (a) six movement parameters computed by rigid body translation and rotation during preprocessing, (b) mean whole brain signal, (c) mean brain signal within the lateral ventricles, and (d) the mean signal within a deep white matter ROI. Inclusion of the first temporal derivatives of these regressors within the linear model accounted for the time‐shifted versions of spurious variance. Correlation coefficients were converted to normally distributed *Z*‐scores using the Fisher transformation and group‐averaged results were reported as voxel‐wise Z‐scores.

Global signal regression (GSR) was included in the primary analysis as it has been shown to improve anatomical specificity, correspondence to anatomical connectivity, and remains the most common processing approach in the rs‐fcMRI literature (Fox, Zhang, Snyder, & Raichle, 2009; Murphy & Fox, 2017). This approach uses a general linear model to regress out the average signal across all voxels in the brain, including physiological noise (e.g., cardiac and respiratory), movement‐related artifact, and nonspecific signals.

To ensure any findings were not unique to the dataset or the pre‐processing strategy we also included an alternate dataset (NKI‐Rockland) that did not rely on GSR (Hwang, Bertolero, Liu, & Esposito, 2017; Nooner et al., 2012). Physiological and other sources of noise were estimated from the MRI data and regressed out together with artifact and movement‐related covariates using a previously described preprocessing pipeline (Hwang et al., 2017). For both datasets the time course of the average BOLD signal within each spherical ROI was compared with the BOLD signal time course of other brain voxels to identify regions with positive or negative correlations.

### 2.3 Evaluation of REM‐activation and REM‐deactivation networks

**FIGURE 1:**
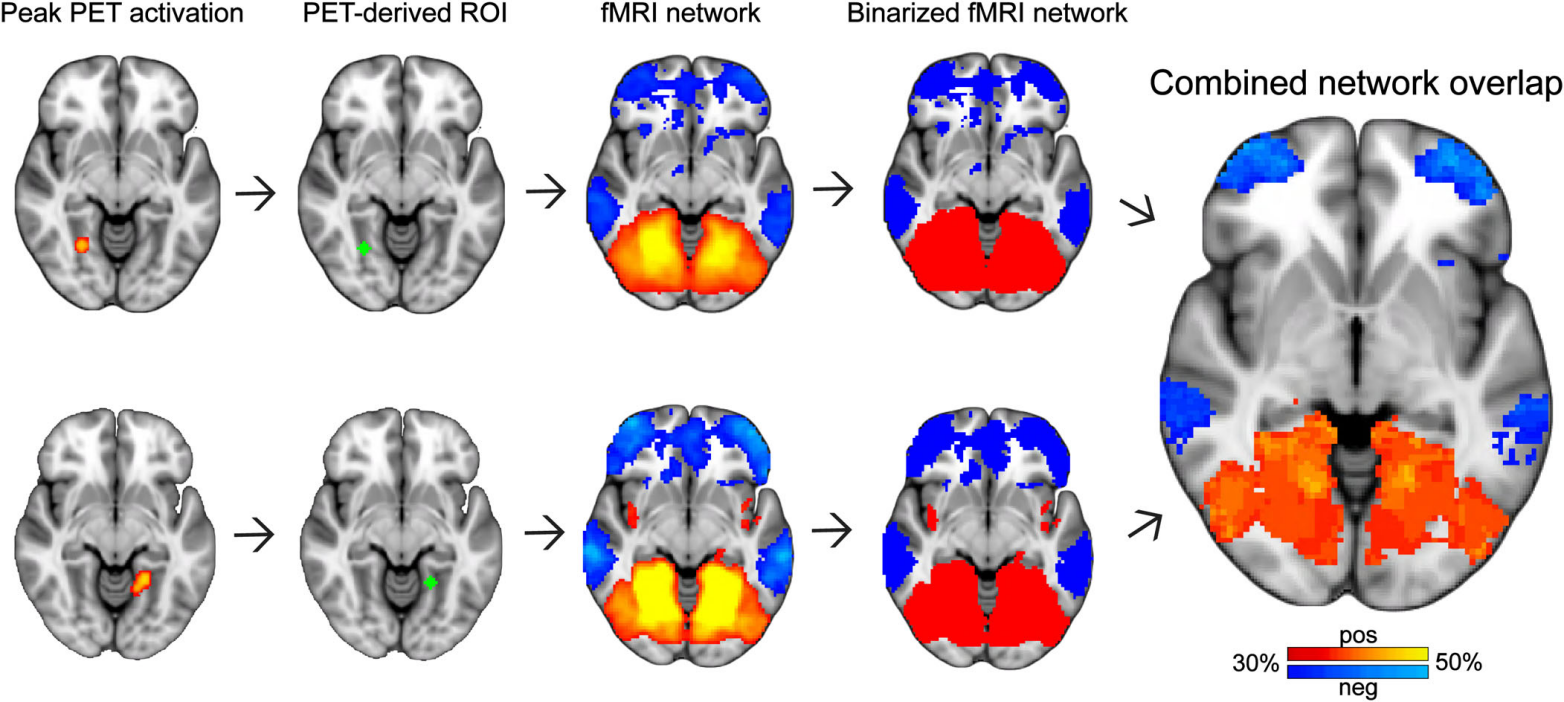
Seed‐based resting state functional connectivity MRI (rs‐fcMRI). Coordinates of peak activations reported from positron emission tomography studies (Column 1) were transformed to MNI152 space as 6 mm spherical regions of interest (ROIs) (Column 2). The brain network associated with each lesion was identified using resting state functional connectivity from a large cohort of normal subjects (Column 3). Positive correlations with the ROI seed are shown in hot colors while negative correlations are shown in cool colors. The positive and negative network derived from each coordinate was thresholded and binarized (Column 4) and all networks were overlapped to identify peak sites where networks overlap across rapid eye movement (REM) activation and deactivation coordinates (Column 5)

Upon generating a voxel‐wise functional connectivity network from each of the PET‐derived coordinates we tested the three hypotheses. We tested whether: significant positive functional connectivity exists within the (a) REM‐activation coordinates and (b) within the REM‐deactivation coordinates, and (c) whether any functional connectivity relationship exists between the REM‐activation and REM‐deactivation coordinates. We also evaluated the spatial organization of sites of network overlap. To visualize common sites of network overlap among the individual ROI‐seeded networks we binarized the network results above a *z*‐score of 8, corresponding to a threshold used previously for visualizing common sites of network overlap (Boes et al., 2015; Fischer et al., 2016; Fox, Buckner, et al., 2012; Laganiere, Boes, & Fox, 2016). An overlap image was generated to visualize areas with multiple overlapping networks as a color‐coded overlap map using Connectome Workbench (https://www.humanconnectome.org/software/connectome-workbench). This process was similar to that used previously for visualizing sites of overlap in lesion network mapping, with PET‐coordinates replacing the lesion masks as seed ROIs (Figure 1) (Boes et al., 2015; Darby, Horn, Cushman, & Fox, 2018). These sites of network overlap were assigned a network affiliation according to the Yeo 17 network parcellation with a naming convention used previously (Schaefer et al., 2018; Yeo et al., 2019).

## 3 RESULTS

First, we report the location of previously reported PET coordinates according to their location within the Yeo functional network parcellation. This showed that REM active regions occurred most frequently in the visual network (18 of 44) and DMN (9 of 44), while REM deactivation coordinates occurred most commonly in the fronto‐parietal network (12 of 30). These data can be found in Table S2 and Figure S2.

### 3.1 REM‐activation and REM‐deactivation networks

**FIGURE 2:**
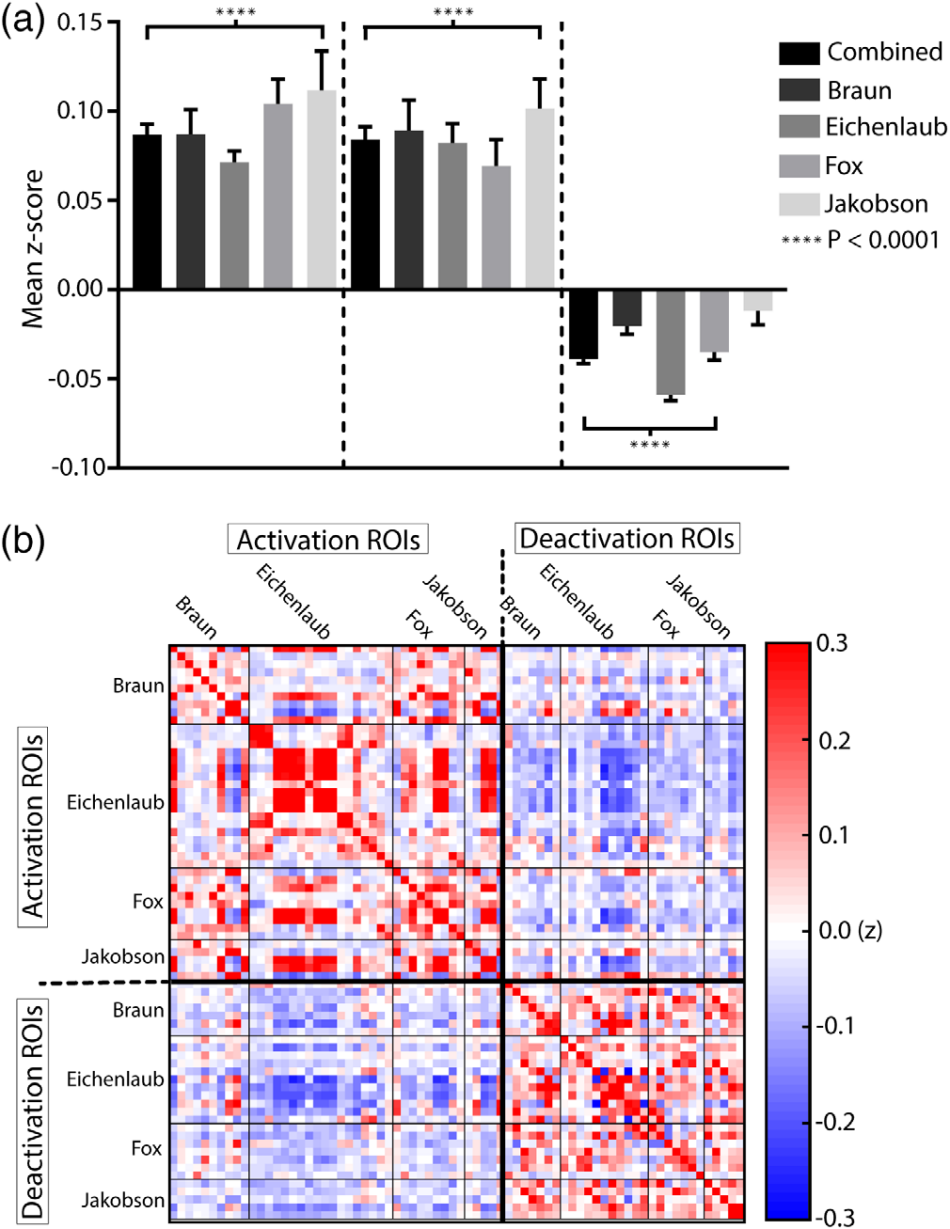
On average the regions of interest (ROIs) within rapid eye movement (REM)‐activation networks were significantly positively correlated (*z* = .09; *p* < .0001). ROIs within the REM‐deactivation networks were also significantly positively correlated (*z* = .08; *p* < .0001), while the relationship between REM‐activation and REM‐deactivation networks was significantly negatively correlated (*z* = −.04; *p* < .0001). (b) The correlation matrix demonstrates this pattern of positive correlations within the REM‐activation and REM‐deactivation networks (shown in red), while negative correlations predominate between these networks (shown in blue)

Next, we evaluated the networks associated with sites of REM activation and deactivation using seed‐based functional connectivity. The BOLD time course within the group of REM‐activation ROIs, was on average, positively correlated, and the same was true of the REM‐deactivation ROIs. Conversely, there was a negative correlation between REM‐activation and REM‐deactivation sites. Figure 2 shows that similar results were observed whether the data was combined across the four studies or when analyzed individually for each of the four studies. REM‐activation peaks were positively correlated (*p* < .0001), as were REM‐deactivation peaks (*p* < .0001), while the relationship between REM‐activation and REM‐deactivation regions were negatively correlated for each study tested (*p* < .001); each shown with a single sample *t* test. The only exception was except the negative correlation between the REM‐activation and for the 5 REM‐deactivation peaks reported in Jakobson, which was not significantly correlated (Mean *z* = −.01, *p* = .14; *χ* ^2^ [2, *N* = 209] = 2.11, *p* = .15). Figure 2b displays a correlation matrix showing that positive correlations exist among the time course data from the REM‐activation and REM‐deactivation regions (in red) while negative correlations (in blue) predominate between REM‐activation and deactivation regions. As a categorical variable the proportion of positive correlations was higher than chance within the REM‐activation and REM‐deactivation networks (*χ* ^2^ [2, *N* = 1,772] = 49.4, *p* < .0001, *χ* ^2^ [2, *N* = 868] = 100.9; *p* < .0001, respectively), while the proportion of negative correlations was higher than chance between REM‐activation and deactivation sites (*χ* ^2^ [2, *N* = 1,260] = 291.5, *p* < .0001).

Since regional overlap of ROIs within the activation and deactivation groups could potentially generate spuriously high positive correlations when combined across analyses, we repeated the combined analysis after removing correlation values from any ROIs pairs occurring within 20 mm of each other in the brain. As a result, 110 REM‐activation ROI pair correlations and 46 REM‐deactivation correlations were removed from the combined group. Consistent with the primary analysis, we found that the proportion of positive correlations within the activation and deactivation groups was each still significantly higher than chance the same pattern of results after removal of 110 REM‐activation pairs and 46 REM‐deactivation pairs that were within close proximity of each other (REM‐activation: *χ* ^2^ (2, *N* = 1,150) = 21.8, *p* < .0001; REM‐deactivation: *χ* ^2^ (2, *N* = 822) = 78.5; *p* < .0001, also) (see Figure S3 correlation matrix). We also repeated the analysis within each individual study after removing any correlation values derived from pairs within 20 mm of each other. The average correlation strength remained significantly positive (*p* < .0001) for all studies individually after removing the regional ROI pairs, with the exception of the activation ROIs from Jakobson, which became significantly negatively correlated (*z* = −.09, *p* < .0001), and the deactivation ROIs from Fox, though statistical power may have contributed to the loss of significance.

Performing the same analysis on non‐GSR processed data showed a similar pattern of network segregation between REM‐activation and deactivation coordinates, but the correlation values were positively shifted relative to the GSR‐processed data and negative correlation values between the REM‐activation and REM‐deactivation sites were no longer present (Figure S3). REM‐activation peaks and REM‐deactivation peaks were each positively correlated within each study (*p* < .0001), while the correlations between the REM‐activation and REM‐deactivation regions showed a significantly weaker correlation (*p* < .0001 for each comparison).

Next, we looked at the spatial topology of sites of overlap among the REM activation and deactivation networks. The sites of maximum overlap from the REM‐activation networks was 22 of 42 networks located in the retrosplenial complex near the parieto‐occipital sulcus (−13, −51, 1.5; default mode C & visual B networks) and parahippocampal gyrus (19, −38, −12.5; visual B network) (Schaefer et al., 2018). Other regions with high overlap included bilateral striate and extrastriate visual cortices on the medial and lateral surface and bilateral medial pulvinar nuclei of the thalamus (Jakab, Blanc, Berenyi, & Szekely, 2012) (visual A & B networks), primary motor cortices (somatomotor A & B networks), as well as medial prefrontal/anterior cingulate cortex and bilateral hippocampus/entorhinal cortex (default mode A & C networks) (Glasser et al., 2017). In contrast, the REM‐deactivation network included the bilateral inferior parietal lobes with a peak overlap of 23 of 30 networks at 48, −55, 52 (fronto‐parietal control B network). Other peaks in this same network included the middle frontal/right orbitofrontal cortices and frontal eye fields (fronto‐parietal control A & C networks). Sites of network overlap also occurred in the right anterior insula and right antero‐ventral thalamus, both nodes of the salience/ventral attention B network (Glasser et al., 2017; Jakab et al., 2012). Table S3 summarizes coordinates corresponding to these networks for REM‐activation and REM‐deactivation along with the network designation.

**FIGURE 3:**
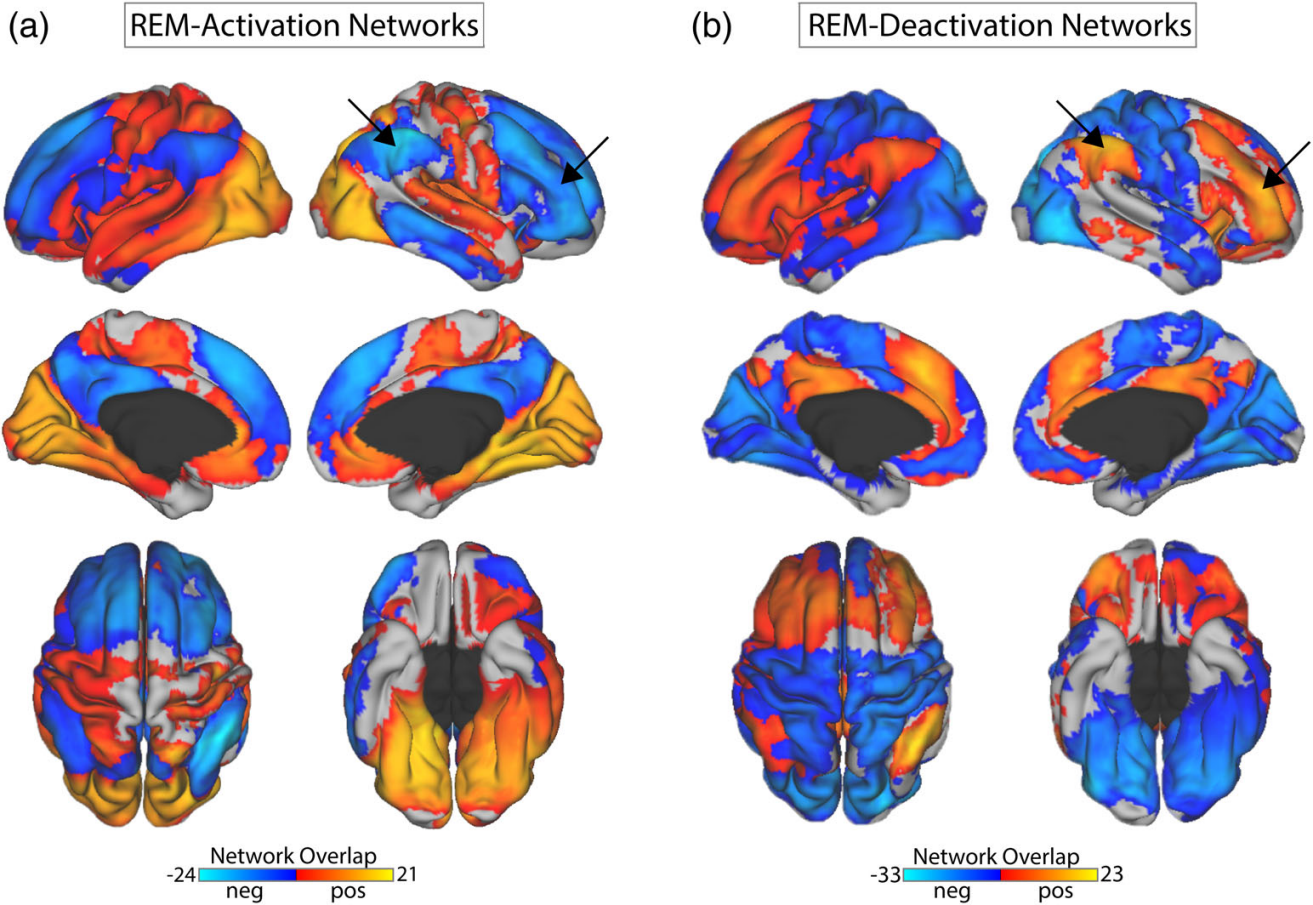
(a) Network overlap map derived from rapid eye movement (REM) activation coordinates. A spherical region of interest (ROI) was created at each peak coordinate reported from previously published positron emission tomography studies. The functional connectivity networks derived from these spherical ROIs were overlapped to visualize common sites of overlap, and the warm and cool color scales display the number of overlapping networks derived from REM activation and deactivation coordinates, respectively. (b) Network overlap map derived from REM deactivation coordinates. Note the similarity between the REM‐activation negative correlations and the REM‐deactivation positive correlations, such as the fronto‐parietal nodes indicated by arrows

Notably the spatial distribution of the regions that were negatively correlated with the REM‐activation network (Figure 3a, cool colors) closely matched the positive correlations of the REM‐deactivation network overlap, and vice versa (Figure 3b, warm colors). Network overlap shown here includes data from all studies combined. Individual network maps were similar across each of the studies evaluated and can be viewed individually (Figure S5).

## 4 DISCUSSION

The regional activation and deactivation patterns associated with REM sleep have been studied in detail over the last 20 years, primarily using PET. Here, we leverage these regional findings and evaluate them in light of normative functional connectivity data from healthy adults resting quietly. Specifically, we generate functional connectivity networks from those brain regions preferentially activated or deactivated during REM sleep and show that, on average, REM‐active areas are functionally connected to the retrosplenial complex and parahippocampal gyrus. Both areas have been implicated in REM sleep previously (Bodizs, Sverteczki, Lazar, & Halasz, 2005; Hong et al., 2009; Nir & Tononi, 2010). Importantly, both regions been implicated in inducing dream‐like states when stimulated electrically (Herbet et al., 2014; Selimbeyoglu & Parvizi, 2010). The most common sites of network overlap were found in the default mode C network and visual networks A & B (Schaefer et al., 2018; Yeo et al., 2011).

The spatial distribution of sites of overlap for the REM‐deactivation networks was quite different and included regions of the dorsolateral prefrontal cortex and parietal cortex. These findings are consistent with prior reports of prefrontal cortex deactivation, but add to this literature by showing an association between these sites and the fronto‐parietal network (Muzur et al., 2002), but we also reported new findings by in showing an association between these sites and the fronto‐parietal network as well as regions of the salience network in the anterior insula and thalamus (also see Maquet et al., 2005). These regional findings raise the interesting possibility to be tested in future studies that differences in the subjective experience between dreaming (intrinsic content that is largely devoid of environmental constraints) and wakefulness (with vigilance toward the environment and reality surveillance) may be supported differentially by these distinct networks. This interpretation would be consistent with the REM‐deactivation network findings overlapping with regions implicated in aspects of behavior that are diminished during dreaming, such as stimulus‐driven reorienting or generating error signals when beliefs are violated (Corbetta & Shulman, 2002; Corlett et al., 2004). It is also possible that these differences in subjective experience in these domains exist on a continuum with wakeful experience, where daydreaming or visual imagery is associated with higher metabolism in the REM activation network and lower metabolism in the REM deactivation network, while the reverse is true in situations requiring vigilance toward the environment.

### 4.1 Functionally segregated versus anticorrelated sleep networks

We observed a negatively correlated relationship between the REM‐activation and deactivation regions when using GSR‐processed data. However, these negative correlations were eliminated when non‐GSR processed data was used for the analysis, as has been reported previously (Murphy, Birn, Handwerker, Jones, & Bandettini, 2009; Murphy & Fox, 2017). The functional significance of negative correlations in GSR‐processed data is unclear and its interpretation is confounded by a mathematical mandate that negative correlations exist after GSR, potentially turning absent correlations into negative correlations when the data are centered at zero following GSR. Despite these issues, GSR does not dictate the spatial organization of negative correlations and it is notable that negative correlations from the REM‐activation network correspond closely with positive correlations from the REM‐deactivation network, and vice versa (Figure 3).

There is emerging, albeit controversial, evidence that brain networks engaged in opposing functional tasks may exhibit negatively correlated activity patterns, such as negatively correlated activity between networks engaged with attention‐demanding tasks versus those active at rest. (Buckner, Andrews‐Hanna, & Schacter, 2008; Hampson, Driesen, Roth, Gore, & Constable, 2010; Kelly, Uddin, Biswal, Castellanos, & Milham, 2008). Similarly, sleep‐ and wake‐promoting regions of the hypothalamus have opposing functions and have negatively correlated activity patterns (Boes et al., 2018). Further study is needed to better understand the functional significance of between‐network negative correlations, including those observed here.

### 4.2 Limitations

There are several limitations to this study. First, the design of this study was to evaluate whether sites of regional activation and deactivation are functionally connected during wakefulness, but we cannot comment on whether these same regions are functionally connected during REM sleep, where the dynamic interactions between brain areas may differ (Chow et al., 2013). Also, while the participants contributing functional connectivity data were instructed to stay awake in the scanner there was no monitoring to ensure they remained awake throughout the imaging acquisition and it is known that individuals often fall asleep during prolonged scans (Hampson et al., 2010; Jakobson et al., 2012). An important follow‐up to the current study will be to evaluate regional REM‐associated PET activation patterns and rs‐fcMRI relationships between these peaks within the same individuals during both wake and REM sleep. Another major caveat to the interpretation of the relationship between REM‐activation and REM‐deactivation ROIs is that negative correlations are only present in the GSR‐processed data, which is commonly the case (Murphy & Fox, 2017). Finally, we allude to potential insights from these results regarding the differences in subjective experience between REM‐associated dreaming and wakefulness. It is important to note that the neural basis of REM sleep can be functionally separated from that of dreaming (Solms, 2000), and the PET studies used to inform our network analyses were from REM sleep and most studies did not specifically assess dreaming, so any extrapolation of our findings on REM sleep to dreaming will require further study. Finally, REM sleep is not homogenous and our results to not differentiate between tonic and phasic subtypes of REM sleep, though the network organization that supports these subtypes could differ.

### 4.3 Future directions

These results generate a number of hypotheses that could be tested in future studies. For example, it is possible that regions identified in the REM‐activation and REM‐deactivation networks may relate to different types of cognitive processing while awake, such as REM‐activation sites being more involved in daydreaming and creative visual imagery versus REM‐deactivation sites being more involved in processes that are lacking or diminished during dreaming, such as reality monitoring, volitional control, and vigilance. It is possible that reduced negative correlation between these networks could have functional relevance for inter‐individual differences in how these subjective experiences are integrated, such that a reduction in the strength of the between network negative correlation may be seen in conditions where dream‐like thought content intrudes into waking life, as has been hypothesized for some forms of hallucinations or psychosis (Lhermitte, 1922). Additionally, given the different neurochemical milieus associated with REM sleep versus wakefulness, it would be interesting to evaluate whether a consistent relationship exists between acetylcholine and monoamine levels and metabolism in these REM‐associated networks.

### 4.4 Conclusion

The current study characterizes the distribution of metabolic changes associated with REM sleep in terms of known functional networks, providing an important extension to previous studies of REM sleep networks (Fox et al., 2013). Our findings are in agreement with prior reports that brain regions preferentially active during REM sleep tend to occur within the DMN, though we observe that sites associated with increased metabolism in REM occur most frequently in visual areas. In contrast, sites with reduced metabolism during REM sleep are associated with the fronto‐parietal and salience networks. Finally, there is a negatively correlated relationship between REM activation and deactivation sites. Taken together, these findings provide insight into the network organization of regional metabolic changes seen in REM sleep and potentially help to inform the contribution of these regions to subjective differences that occur in wake versus REM sleep.

## CONFLICT OF INTEREST

The authors declare no potential conflict of interest.

## Supporting information


**Appendix** **S1**: Supporting Information

## Data Availability

The data that support the findings of this study are available from the corresponding author upon reasonable request.

## References

[hbm25102-bib-0001] Aserinsky, E. , & Kleitman, N. (1953). Regularly occurring periods of eye motility, and concomitant phenomena, during sleep. Science, 118(3062), 273–274. 13089671 10.1126/science.118.3062.273

[hbm25102-bib-0002] Avants, B. B. , Tustison, N. J. , Song, G. , Cook, P. A. , Klein, A. , & Gee, J. C. (2011). A reproducible evaluation of ANTs similarity metric performance in brain image registration. NeuroImage, 54(3), 2033–2044. 10.1016/j.neuroimage.2010.09.025 20851191 PMC3065962

[hbm25102-bib-0003] Bodizs, R. , Sverteczki, M. , Lazar, A. S. , & Halasz, P. (2005). Human parahippocampal activity: Non‐REM and REM elements in wake–sleep transition. Brain Research Bulletin, 65, 169–176. 10.1016/j.brainresbull.2005.01.002 15763184

[hbm25102-bib-0004] Boes, A. D. , Fischer, D. , Geerling, J. C. , Bruss, J. , Saper, C. B. , & Fox, M. D. (2018). Connectivity of sleep‐ and wake‐promoting regions of the human hypothalamus observed during resting wakefulness. Sleep, 7, 1–12. 10.1093/sleep/zsy108 PMC6454456 29850898

[hbm25102-bib-0005] Boes, A. D. , Prasad, S. , Liu, H. , Liu, Q. , Pascual‐Leone, A. , Caviness, V. S. , & Fox, M. D. (2015). Network localization of neurological symptoms from focal brain lesions. Brain: A Journal of Neurology, 138(Pt 10, 3061–3075. 10.1093/brain/awv228 26264514 PMC4671478

[hbm25102-bib-0006] Braun, A. R. , Balkin, T. J. , Wesensten, N. J. , Carson, R. E. , Varga, M. , Baldwin, P. , … Herscovitch, P. (1997). Regional cerebral blood flow throughout the sleep–wake cycle: An H2(15)O PET study. Brain, 120(Pt 7, 1173–1197. 9236630 10.1093/brain/120.7.1173

[hbm25102-bib-0007] Buckner, R. , Andrews‐Hanna, J. , & Schacter, D. (2008). The Brain's default network: Anatomy, function, and relevance to disease. Annals of the new York Academy of Sciences, 38, 1–38. 10.1196/annals.1440.011 18400922

[hbm25102-bib-0008] Chow, H. M. , Horovitz, S. G. , Carr, W. S. , Picchioni, D. , Coddington, N. , Fukunaga, M. , … Braun, A. R. (2013). Rhythmic alternating patterns of brain activity distinguish rapid eye movement sleep from other states of consciousness. Proceedings of the National Academy of Sciences of the United States of America, 110(25), 110–10305. 10.1073/pnas.1217691110 PMC3690889 23733938

[hbm25102-bib-0009] Cicogna, P. , Natale, V. , Occhionero, M. , & Bosinelli, M. (2000). Slow wave and REM sleep mentation. Sleep Research Online, 3(2), 67–72. 11382903

[hbm25102-bib-0010] Corbetta, M. , & Shulman, G. L. (2002). Control of goal‐directed and stimulus‐driven attention in the brain. Nature Reviews. Neuroscience, 3, 201–215. 10.1038/nrn755 11994752

[hbm25102-bib-0011] Corlett, P. R. , Aitken, M. R. F. , Dickinson, A. , Shanks, D. R. , Honey, G. D. , Honey, R. A. E. , … Fletcher, P. C. (2004). Prediction error during retrospective revaluation of causal associations in humans: fMRI evidence in favor of an associative model of learning. Neuron, 44, 877–888. 15572117 10.1016/j.neuron.2004.11.022

[hbm25102-bib-0012] Darby, R. , Horn, A. , Cushman, F. , & Fox, M. D. (2018). Lesion network localization of criminal behavior. Proceedings of the National Academy of Sciences of the United States of America, 115(3), 3–8. 29255017 10.1073/pnas.1706587115 PMC5776958

[hbm25102-bib-0013] Domhoff, G. W. , & Fox, K. C. R. (2015). Dreaming and the default network: A review, synthesis, and counterintuitive research proposal. Consciousness and Cognition, 33, 342–353. 10.1016/j.concog.2015.01.019 25723600

[hbm25102-bib-0014] Eichenlaub, J. , Nicolas, A. , Daltrozzo, J. , Redoute, J. , Costes, N. , & Ruby, P. (2014). Resting brain activity varies with dream recall frequency between subjects. Neuropsychopharmacology, 39(7), 1594–1602. 10.1038/npp.2014.6 24549103 PMC4023156

[hbm25102-bib-0015] Fischer, D. , Boes, A. , Demertzi, A. , Evrard, H. , Laureys, S. , Edlow, B. , … Geerling, J. (2016). A human brain network based on coma‐causing brainstem lesions. Neurology, 87(23), 2427–2434. 27815400 10.1212/WNL.0000000000003404 PMC5177681

[hbm25102-bib-0016] Fox, K. C. R. , Nijeboer, S. , Solomonova, E. , Domhoff, G. , & Christoff, K. (2013). Dreaming as mind wandering: Evidence from functional neuroimaging and first‐person content reports. Frontiers in Human Neuroscience, 7, 412 10.3389/fnhum.2013.00412 23908622 PMC3726865

[hbm25102-bib-0017] Fox, M. D. , Buckner, R. L. , White, M. P. , Greicius, M. D. , & Pascual‐Leone, A. (2012). Efficacy of transcranial magnetic stimulation targets for depression is related to intrinsic functional connectivity with the subgenual cingulate. Biological Psychiatry, 72, 595–603. 22658708 10.1016/j.biopsych.2012.04.028 PMC4120275

[hbm25102-bib-0018] Fox, M. D. , Halko, M. A. , Eldaief, M. C. , & Pascual‐Leone, A. (2012). Measuring and manipulating brain connectivity with resting state functional connectivity magnetic resonance imaging (fcMRI) and transcranial magnetic stimulation (TMS). NeuroImage, 62(4), 2232–2243. 22465297 10.1016/j.neuroimage.2012.03.035 PMC3518426

[hbm25102-bib-0019] Fox, M. D. , Snyder, A. Z. , Vincent, J. L. , Corbetta, M. , Van Essen, D. C. , & Raichle, M. E. (2005). The human brain is intrinsically organized into dynamic, anticorrelated functional networks. Proceedings of the National Academy of Sciences of the United States of America, 102(27), 9673–9678. 10.1073/pnas.0504136102 15976020 PMC1157105

[hbm25102-bib-0020] Fox, M. D. , Zhang, D. , Snyder, A. Z. , & Raichle, M. E. (2009). The global signal and observed anticorrelated resting state brain networks. Journal of Neurophysiology, 101(6), 3270–3283. 10.1152/jn.90777.200890777.2008 pii. 19339462 PMC2694109

[hbm25102-bib-0021] Glasser, M. F. , Coalson, T. S. , Robinson, E. C. , Hacker, C. D. , Yacoub, E. , Ugurbil, K. , … Van, D. C. (2017). A multi‐modal parcellation of human cerebral cortex. Nature, 536(7615), 171–178. 10.1038/nature18933.A PMC4990127 27437579

[hbm25102-bib-0022] Grabner, G. , Janke, A. , Budge, M. , Smith, D. , Pruessner, J. , & Collins, D. (2006). Symmetric atlasing and model based segmentation: An application to hte hippocampus in older adults. Medical Image Computing and Computer‐Assisted Intervention, 9, 58–66. 10.1007/11866763_8 17354756

[hbm25102-bib-0023] Hampson, M. , Driesen, N. , Roth, J. K. , Gore, J. C. , & Constable, R. T. (2010). Functional connectivity between task‐positive and task‐negative brain areas and its relation to working memory performance. Magnetic Resonance Imaging, 28(8), 1051–1057. 10.1016/j.mri.2010.03.021 20409665 PMC2936669

[hbm25102-bib-0024] Herbet, G. , Lafargue, G. , Menjot, N. , Champ, D. , Moritz‐gasser, S. , Bonnetblanc, F. , & Duffau, H. (2014). Disrupting posterior cingulate connectivity disconnects consciousness from the external environment. Neuropsychologia, 56, 239–244. 10.1016/j.neuropsychologia.2014.01.020 24508051

[hbm25102-bib-0025] Holmes, A. J. , Hollinshead, M. O. , O'Keefe, T. M. , Petrov, V. I. , Fariello, G. R. , Wald, L. L. , … Buckner, R. L. (2015). Brain genomics superstruct project initial data release with structural, functional, and behavioral measures. Scientific Data, 2, 1–16. 10.1038/sdata.2015.31 PMC4493828 26175908

[hbm25102-bib-0026] Hong, C. C. , Harris, J. C. , Pearlson, G. D. , Kim, J. , Calhoun, V. D. , Fallon, J. H. , … Pekar, J. (2009). fMRI evidence for multisensory recruitment associated with rapid eye movements during sleep. Human Brain Mapping, 1722, 1705–1722. 10.1002/hbm.20635 PMC2753360 18972392

[hbm25102-bib-0027] Hwang, K. , Bertolero, M. A. , Liu, W. B. , & Esposito, M. D. (2017). The human thalamus is an integrative hub for functional brain networks. Journal of Neuroscience, 37(23), 5594–5607. 10.1523/JNEUROSCI.0067-17.2017 28450543 PMC5469300

[hbm25102-bib-0028] Jakab, A. , Blanc, R. , Berenyi, E. L. , & Szekely, G. (2012). Generation of individualized thalamus target maps by using statistical shape models and thalamocortical tractography. American Journal of Neuroradiology, 33(11), 2110–2116. 10.3174/ajnr.A3140 22700756 PMC7965575

[hbm25102-bib-0029] Jakobson, A. J. , Laird, A. R. , Maller, J. J. , Conduit, R. D. , & Fitzgerald, P. B. (2012). Brain activity in sleep compared to wakefulness: A meta‐analysis. Journal of Behavioral and Brain Sciences, 2(5), 249–257.

[hbm25102-bib-0030] Kelly, A. , Uddin, L. , Biswal, B. , Castellanos, F. , & Milham, M. (2008). Competition between functional brain networks mediates behavioral variability. NeuroImage, 39, 527–537. 10.1016/j.neuroimage.2007.08.008 17919929

[hbm25102-bib-0031] Laganiere, S. , Boes, A. D. , & Fox, M. D. (2016). Network localization of hemichorea‐hemiballismus. Neurology, 86(23), 2187–2195. 10.1212/WNL.0000000000002741 27170566 PMC4898318

[hbm25102-bib-0032] Lhermitte, J. (1922). Syndrome de la calotte du pedoncule cerebral: Les troubles psycho‐sensoriels dans les lesions du mesocephale. Revista de Neurologia, 38, 1359–1366.

[hbm25102-bib-0033] Lövblad, K. , Thomas, R. , Jakob, P. , Scammell, T. , Bassetti, C. , Griswold, M. , … Warach, S. (1999). Silent functional magnetic resonance imaging demonstrates focal activation in rapid eye movement sleep. Neurology, 53(9), 2195. 10.1212/wnl.53.9.2193 10599807

[hbm25102-bib-0034] Maquet, P. , Laureys, S. , Peigneux, P. , Fuchs, S. , Petiau, C. , Phillips, C. , … Cleeremans, A. (2000). Experience‐dependent changes in cerebral activation during human REM sleep. Nature Neuroscience, 3(8), 831–836. 10903578 10.1038/77744

[hbm25102-bib-0035] Maquet, P. , Ruby, P. , Maudoux, A. , Albouy, G. , Sterpenich, V. , Dang‐vu, T. , … Laureys, S. (2005). Human cognition during REM sleep and the activity profile within frontal and parietal cortices: A reappraisal of functional neuroimaging data. Progress in Brain Research, 150, 219–227. 10.1016/S0079-6123(05)50016-5 16186026

[hbm25102-bib-0036] Miyauchi, S. , Misaki, M. , Kan, S. , Fukunaga, T. , & Koike, T. (2009). Human brain activity time‐locked to rapid eye movements during REM sleep. Experimental Brain Research, 192, 657–667. 10.1007/s00221-008-1579-2 18830586

[hbm25102-bib-0037] Murphy, K. , Birn, R. M. , Handwerker, D. A. , Jones, T. B. , & Bandettini, P. A. (2009). The impact of global signal regression on resting state correlations: Are anti‐correlated networks introduced? NeuroImage, 44(3), 893–905. 10.1016/j.neuroimage.2008.09.036 18976716 PMC2750906

[hbm25102-bib-0038] Murphy, K. , & Fox, M. (2017). Towards a consensus regarding global signal regression for resting state functional connectivity MRI. NeuroImage, 154, 169–173. 10.1016/j.neuroimage.2016.11.052 27888059 PMC5489207

[hbm25102-bib-0039] Muzur, A. , Pace‐Schott, E. F. , & Hobson, J. A. (2002). The prefrontal cortex in sleep. Trends in Cognitive Sciences, 6(11), 475–481. 12457899 10.1016/s1364-6613(02)01992-7

[hbm25102-bib-0040] Nir, Y. , & Tononi, G. (2010). Dreaming and the brain: From phenomenology to neurophysiology. Trends in Cognitive Sciences, 14(2), 88–100. https://doi.org/10.1016/j.tics.2009.12.001 20079677 10.1016/j.tics.2009.12.001 PMC2814941

[hbm25102-bib-0041] Nofzinger, E. A. (1997). Forebrain activation in REM sleep: An FDG PET study. Brain Research, 770, 192–201. 9372219 10.1016/s0006-8993(97)00807-x

[hbm25102-bib-0042] Nooner, K. B. , Colcombe, S. J. , Tobe, R. H. , Mennes, M. , Benedict, M. M. , Moreno, A. L. , … Michael, P. (2012). The NKI‐Rockland sample: A model for accelerating the pace of discovery science in psychiatry. Frontiers in Neuroscience, 6, 1–11. 10.3389/fnins.2012.00152 23087608 PMC3472598

[hbm25102-bib-0043] Saper, C. B. , & Fuller, P. M. (2017). Wake–sleep circuitry: An overview. Current Opinion in Neurobiology, 44, 186–192. 10.1016/j.conb.2017.03.021 28577468 PMC5531075

[hbm25102-bib-0044] Schaefer, A. , Kong, R. , Gordon, E. M. , Laumann, T. O. , Zuo, X. , Holmes, A. J. , … Yeo, T. B. (2018). Local‐global parcellation of the human cerebral cortex from intrinsic functional connectivity MRI. Cerebral Cortex, 28(9), 3095–3114. 10.1093/cercor/bhx179 28981612 PMC6095216

[hbm25102-bib-0045] Schwartz, J. R. L. , & Roth, T. (2008). Neurophysiology of sleep and wakefulness: Basic science and clinical implications. Current Neuropharmacology, 6(4), 367–378. 10.2174/157015908787386050 19587857 PMC2701283

[hbm25102-bib-0046] Selimbeyoglu, A. , & Parvizi, J. (2010). Electrical stimulation of the human brain: Perceptual and behavioral phenomena reported in the old and new literature. Frontiers in Human Neuroscience, 4, 1–11. 10.3389/fnhum.2010.00046 20577584 PMC2889679

[hbm25102-bib-0047] Siclari, F. , Baird, B. , Perogamvros, L. , Bernardi, G. , LaRocque, J. J. , Riedner, B. , … Tononi, G. (2017). The neural correlates of dreaming. Nature Neuroscience, 20(6), 872–878. 10.1038/nn.4545 28394322 PMC5462120

[hbm25102-bib-0048] Solms, M. (2000). Dreaming and REM sleep are controlled by different brain mechanisms. Behavioral and Brain Sciences, 23, 790–1121. 10.1017/s0140525x00003988 11515144

[hbm25102-bib-0049] Van Dijk, K. R. , Hedden, T. , Venkataraman, A. , Evans, K. C. , Lazar, S. W. , & Buckner, R. L. (2010). Intrinsic functional connectivity as a tool for human connectomics: Theory, properties, and optimization. Journal of Neurophysiology, 103(1), 297–321. 10.1152/jn.00783.2009 19889849 PMC2807224

[hbm25102-bib-0050] Van Essen, D. (2002). Windows on the brain: The emerging role of atlases and databases in neuroscience. Current Opinion in Neurobiology, 12, 574–579. 12367638 10.1016/s0959-4388(02)00361-6

[hbm25102-bib-0051] Wehrle, R. , Czisch, M. , Kaufmann, C. , Wetter, T. C. , Holsboer, F. , Auer, D. P. , & Ca, T. P. (2005). Rapid eye movement‐related brain activation in human sleep: A functional magnetic resonance imaging study. Neuroreport, 16(8), 53–57. 15891584 10.1097/00001756-200505310-00015

[hbm25102-bib-0052] Wu, C. W. , Liu, P. , Tsai, P. , Wu, Y. , Hung, C. , Tsai, Y. , … Lin, C. (2012). Variations in connectivity in the sensorimotor and default‐mode networks during. Brain Connectivity, 2(January 2014), 177–190. 10.1089/brain.2012.0075 22817652

[hbm25102-bib-0053] Yeo, B. T. T. , Krienen, F. M. , Sepulcre, J. , Sabuncu, M. R. , Lashkari, D. , Hollinshead, M. , … Buckner, R. L. (2011). The organization of the human cerebral cortex estimated by intrinsic functional connectivity. Journal of Neurophysiology, 106, 1125–1165. 10.1152/jn.00338.2011 21653723 PMC3174820

