# Supplementary material for: Rapid eye movement sleep patterns of brain activation and deactivation occur within unique functional networks
Source: Hum Brain Mapp. 2020 Jun 23;41(14):3984–92. doi: 10.1002/hbm.25102 (PMC7469766; doi:10.1002/hbm.25102)
Supplement: Supplementary file 1 — Appendix S1: Supporting Information [file HBM-41-3984-s001.pdf]

SUPPORTING INFORMATION

for

REM sleep patterns of brain activation and deactivation occur within unique functional networks

Running Title: REM sleep networks

Brandt D. Uitermarkt, MA [a] *

Joel Bruss, BA [a]

Kai Hwang, PhD [b]

Aaron D. Boes, MD, PhD [a]

*Send correspondence to:
Brandt D. Uitermarkt, MA
Iowa Neuroimaging & Noninvasive Brain Stimulation Laboratory
Departments of Pediatrics, Neurology, & Psychiatry
University of Iowa Hospitals and Clinics
W276 GH, 200 Hawkins Drive, Iowa City, IA 52242
email: brandt-uitermarkt@uiowa.edu

[a] Neuroimaging & Noninvasive Brain Stimulation Laboratory, Departments of Pediatrics, Neurology & Psychiatry, University of Iowa Hospitals and Clinics, Mail Code: W276 GH, Iowa City, IA, 52242

[b] Hwang Laboratory for Neurocognitive Dynamics, Department of Psychological and Brain Sciences, University of Iowa Hospitals and Clinics, Mail Code: E11 Seashore Hall, Iowa City, IA, 52242

REM Activation Coordinates						REM Deactivation Coordinates					
Talairach (x, y, z]			MNI (x, y, z)			Talairach (x, y, z)			MNI (x, y, z)		
**Braun et al. (1997)**											
18	-46	-12	21	-49	-14	28	-30	20	32	-28	20
-2	-18	-24	-1	-20	-29	-6	-32	28	-5	-30	30
-14	-28	-16	-14	-30	-19	-30	38	-12	-32	41	-22
-4	18	8	-3	21	2	44	32	20	49	38	13
-2	18	-20	-1	19	-29	46	18	8	51	22	1
-16	-26	-20	-16	-28	-24	54	-54	24	60	-53	27
24	-40	-8	27	-42	-10	46	-42	24	51	-41	25
4	30	0	5	33	-8						
-2	44	24	-1	51	17						
24	-40	-12	27	-42	-14						
**Eichenlaub et al. (2014)**											
-3	-25	58	-2	-22	63	11	39	-24	12	44	-27
-29	-28	59	-28	-25	66	-7	21	42	-6	27	43
-39	-16	14	-40	-13	15	25	26	-21	27	30	-23
29	-84	15	30	-84	19	-29	39	-15	-30	44	-18
-36	-80	0	-38	-81	3	45	10	13	48	14	9
-31	-79	16	-33	-79	21	-41	-67	39	-42	-66	46
-14	-53	-5	-16	-52	-5	53	-48	36	57	-46	40
30	-30	-22	32	-28	-23	-53	-52	36	-54	-51	42
-22	-70	-13	-24	-70	-11	-5	-62	28	-4	-61	34
6	-84	19	6	-84	25	-1	-28	31	0	-24	30
16	-88	19	16	-88	24	32	15	3	34	21	1
51	-16	5	54	-15	3						
-59	-17	3	-62	-15	3						
41	-51	-14	44	-49	-15						
1	2	34	2	6	33						
34	-16	3	36	-15	3						
19	-6	-17	21	-4	-18						
-1	-9	-13	-2	-7	-15						
**Fox et al. (2013)**											
2	32	2	1	28	9	-8	-34	28	-9	-36	26
28	-66	4	25	-64	2	32	44	20	28	37	26
24	-40	-10	21	-38	-8	-46	26	-2	-44	23	4
-16	-26	-18	-16	-24	-15	-32	38	-10	-30	35	-2
18	-30	-6	16	-29	-4	38	36	-12	34	33	-3
-18	-50	-8	-18	-48	-8	18	46	-14	16	42	-4
22	-58	-6	19	-55	-6	28	-42	20	25	-43	19
22	-18	-14	19	-17	-10						
8	-14	-18	7	-13	-13						
**Jakobson et al. (2012)**											
-2	20	8	-3	16	13	28	42	20	25	35	26
4	30	0	3	26	7	-32	38	-10	-30	35	-2
20	-46	-10	17	-44	-9	42	12	22	38	7	25
24	-42	-8	21	-40	-6	24	50	4	21	44	12
2	40	12	1	34	18	22	54	-2	19	49	7

Supplementary Table 1. Peak rapid eye movement (REM)-activation and REM-deactivation coordinates reported for each study in Talairach (x,y,z) and MNI152 (x,y,z) coordinate space.

	Study	Visual	Somato motor	Dorsal Attn.	Ventral Attn.	Limbic	Fronto-parietal	DMN	Unclassified
**REM Activation ROIs**	Combined	19	5	1	2	2	0	9	6
	Braun	3	0	0	0	1	0	4	2
	Eichenlaub	9	5	1	2	0	0	0	2
	Fox	5	0	0	0	0	0	3	1
	Jakobson	2	0	0	0	0	0	2	1
**REM Deactivation ROIs**	Combined	2	1	1	2	3	12	7	2
	Braun	1	1	0	0	0	1	4	0
	Eichenlaub	1	0	0	2	2	6	0	0
	Fox	0	0	0	0	1	2	2	2
	Jakobson	0	0	1	0	0	3	1	0

Supplementary Table 2. Rapid eye movement **(**REM) activation and deactivation coordinates are classified according to their location within the Yeo 7 functional network parcellation (Yeo, 2011), which is also shown in Supplemental Figure 2.

REM-Activation Peak Network Overlap				
**Region**	**Network(s)**	**MNI (x,y,z)**		
Retrosplenial cortex	default mode C	-13	-51	1.5
		15	-43	1
Parahippocampal gyrus	visual B	19	-38	-12.5
		-20	-44	-13
Visual cortex	visual A & B	13	-59	-2
		-17	-60	-4
		47	-65	-1
		-43	-85	-3
Thalamus (medial pulvinar nuclei)	visual A & B	17	-29	0
		-18	-29	3
Motor cortex	somatomotor A	49	-8	51
		-42	-12	51
Hippocampus / Entorhinal cortex	default mode A & C	26	-15	-19
		-24	-17	-19
Medial prefrontal cortex	default mode A	3	46	-7
		-7	45	-7
Anterior cingulate cortex	default mode A	4	37	2
		-3	37	2

REM-Deactivation Peak Network Overlap				
**Region**	**Network(s)**	**MNI (x,y,z)**		
Inferior parietal cortex	fronto-parietal control B	48	-55	52
		-46	-55	51
Middle frontal cortex	fronto-parietal control B	39	55	7
		-37	54	8
Right orbitofrontal cortex	fronto-parietal control B	45	49	-11
Frontal eye fields	fronto-parietal control B	6	33	36
		-2	29	40
		45	24	40
Right anterior insula	salience/ventral attention C	31	22	-9
		-37	17	-4
Right antero-ventral thalamus	salience/ventral attention C	11	-8	3

Supplementary Table 3. The x,y,z MNI152 coordinates for sites of overlap from rapid eye movement (REM)-activation and REM-deactivation networks are included with the associated functional connectivity network.

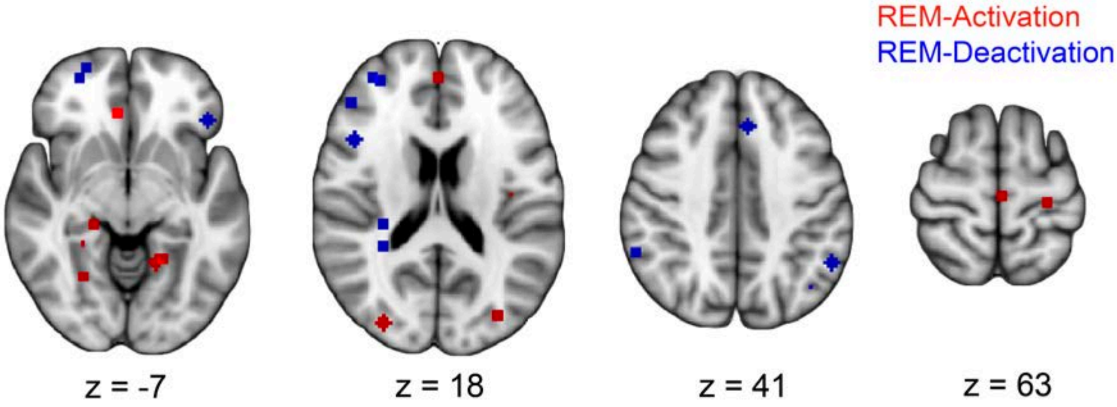

Supplementary Figure 1. 4 selected axial slices depict the spatial distribution of positron emission tomography-derived 6mm spherical regions of interest (ROIs) used as seeds in the resting state functional connectivity network analysis. Red spheres represent rapid eye movement (REM)-activation ROIs and blue spheres represent REM-deactivation ROIs.

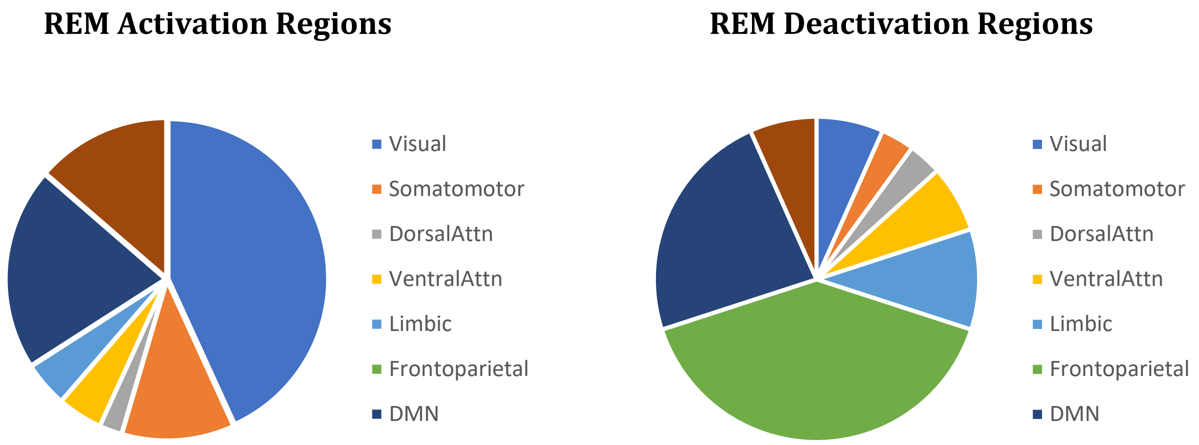

Supplementary Figure 2. Rapid eye movement (REM) Activation and Deactivation Coordinates Plotted According to Functional Brain Networks. The number of REM activation and deactivation regions-of-interest (ROIs) are classified according to their distribution within the Yeo 7 functional networks, also reported in Supplementary Table 2.

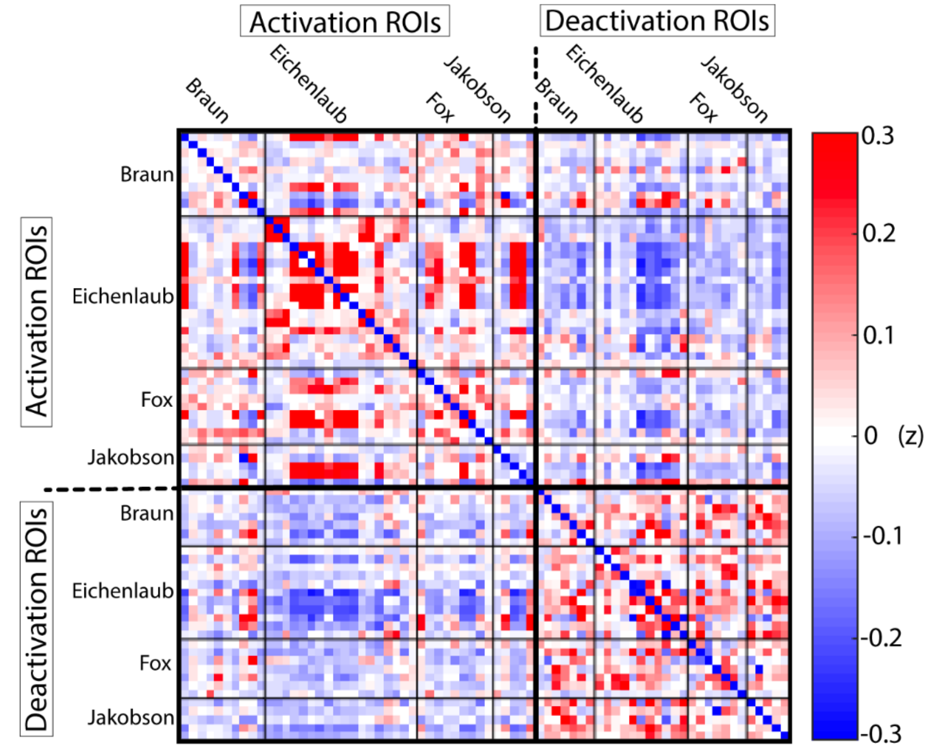

Supplementary Figure 3. The correlation matrix displays the strength of functional connectivity between spherical regions of interest (ROIs) included in the main analysis (see Figure 2), except all ROI pairs occurring within 20mm of each other have been assigned a correlation value of 0 to evaluate the possible confound that close spatial proximity may account for the positive correlations observed amongst the REM activation and REM deactivation regions. The proportion of positive correlations within the activation and deactivation groups was each still significantly higher than chance (REM-activation: χ^2 (2, N = 1,150) = 21.8, P < .0001; REM-deactivation: χ^2 (2, N = 822) = 78.5 ; P < .0001).

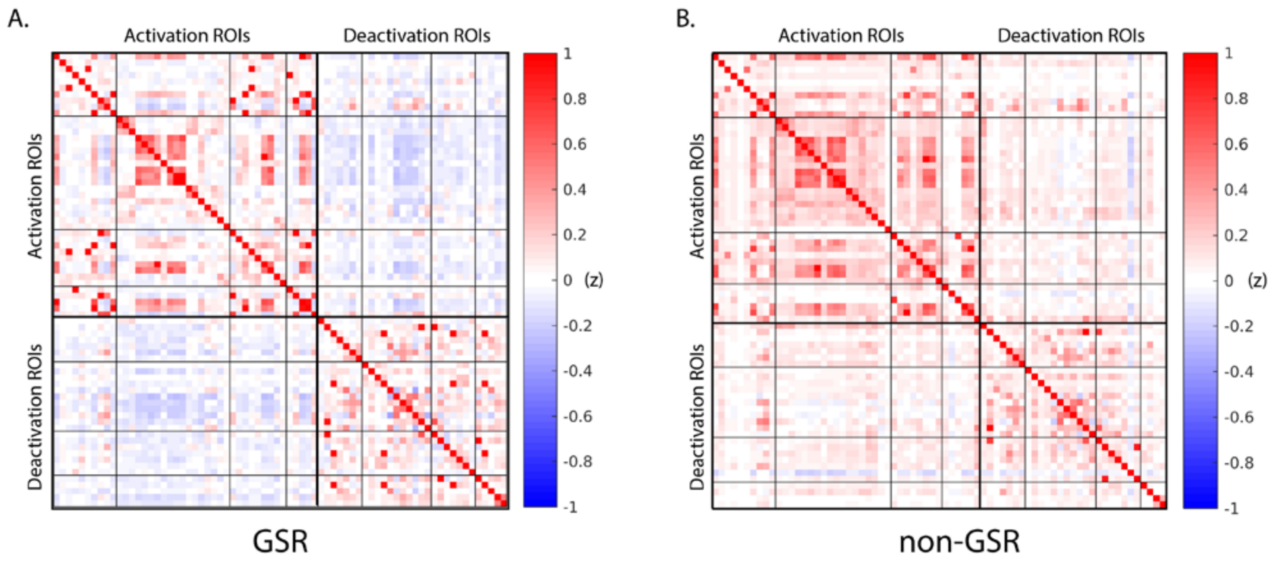

Supplementary Figure 4. The correlation matrices display the strength of functional connectivity between spherical regions of interest (ROIs) located at sites of rapid eye movement (REM)-activation and REM-deactivation. A shows the correlation matrix for the primary analysis using the dataset with global signal regression (GSR). B shows the dataset without GSR. Note that the results are positively shifted and very few negative correlations are present.

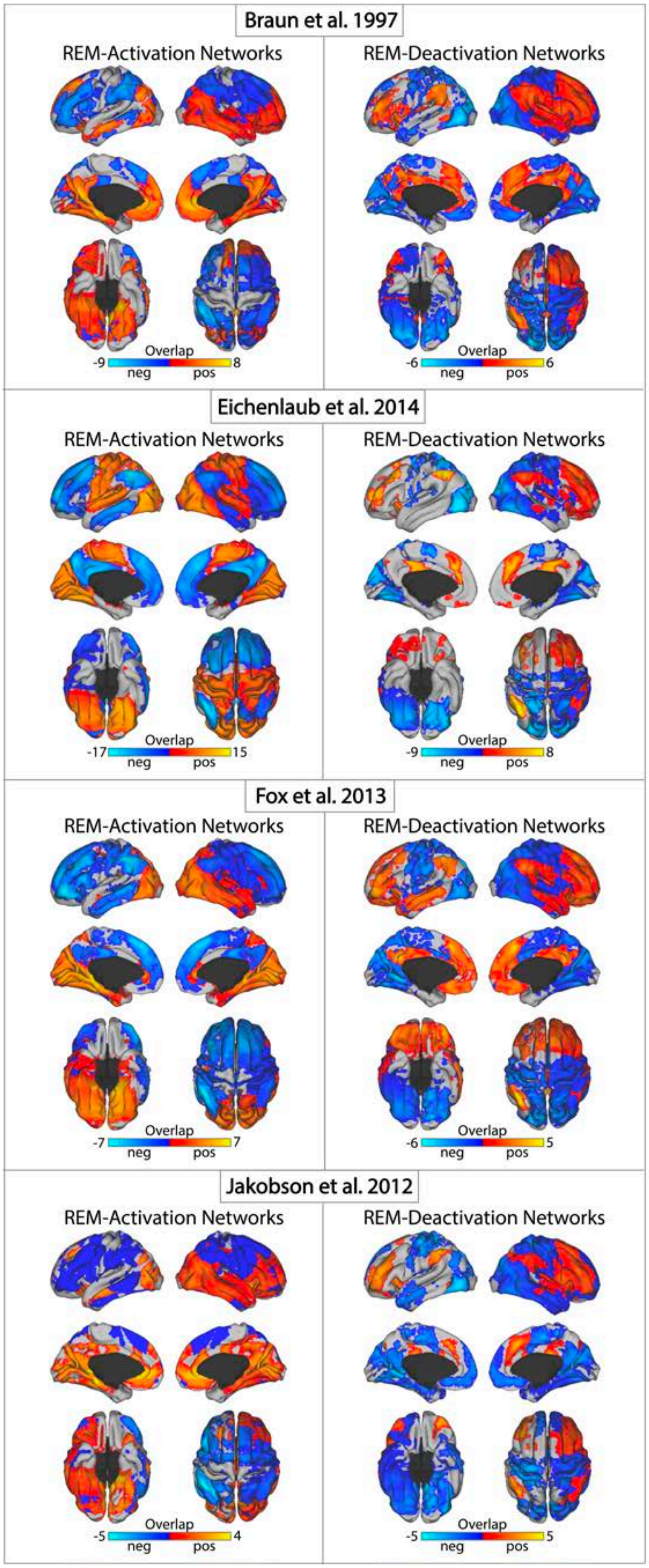

Braun et al. 1997
REM-Activation Networks
REM-Deactivation Networks
Overlap
-9 neg pos 8
-6 neg pos 6
Eichenlaub et al. 2014
REM-Activation Networks
REM-Deactivation Networks
Overlap
-17 neg pos 15
-9 neg pos 8
Fox et al. 2013
REM-Activation Networks
REM-Deactivation Networks
Overlap
-7 neg pos 7
-6 neg pos 5
Jakobson et al. 2012
REM-Activation Networks
REM-Deactivation Networks
Overlap
-5 neg pos 4
-5 neg pos 5

Supplementary Figure 5. This figure displays a network overlap map of rapid eye movement (REM) activation and deactivation sites from coordinates in each individual study that contributed to the analysis. A spherical region of interest (ROI) was created at each peak coordinate reported from previously published positron emission tomography (PET) studies. The functional connectivity networks derived from these spherical ROIs were overlapped to visualize common sites of overlap amongst positive and negative correlation maps, which are shown in warm and cool color scales, respectively.
